# Harmonizing 10,000 connectomes: site-invariant representation learning for multi-site analysis of network connectivity and cognitive impairment

**DOI:** 10.1117/1.JMI.12.6.064001

**Published:** 2025-11-05

**Authors:** Nancy R. Newlin, Michael E. Kim, Praitayini Kanakaraj, Elyssa McMaster, Chloe Cho, Chenyu Gao, Timothy J. Hohman, Lori Beason-Held, Susan M. Resnick, Sid E. O’Bryant, Nicole Phillips, Robert C. Barber, David A. Bennett, Lisa L. Barnes, Sarah Biber, Sterling Johnson, Derek Archer, Zhiyuan Li, Lianrui Zuo, Daniel Moyer, Bennett A. Landman

**Affiliations:** a Vanderbilt University, Department of Computer Science, Nashville, Tennessee, United States; b Vanderbilt University, Department of Electrical and Computer Engineering, Nashville, Tennessee, United States; c Vanderbilt University, Department of Biomedical Engineering, Nashville, Tennessee, United States; d Vanderbilt University Medical Center, Vanderbilt Memory and Alzheimer’s Center, Nashville, Tennessee, United States; e Vanderbilt University Medical Center, Vanderbilt Genetics Institute, Nashville, Tennessee, United States; f National Institute on Aging, National Institutes of Health, Laboratory of Behavioral Neuroscience, Baltimore, Maryland, United States; g University of North Texas Health Science Center, Institute for Translational Research, Fort Worth, Texas, United States; h Rush University Medical Center, Rush Alzheimer’s Disease Center, Chicago, Illinois, United States; i University of Washington, National Alzheimer’s Coordinating Center, Seattle, Washington United States; j University of Wisconsin, School of Medicine and Public Health, Wisconsin Alzheimer’s Disease Research Center, Madison, Wisconsin, United States; k Vanderbilt University, Department of Neurology, Nashville, Tennessee, United States; l Vanderbilt University, Vanderbilt University Institute of Imaging Science, Nashville, Tennessee, United States

**Keywords:** multi-site, diffusion imaging, connectomics, brain networks, machine learning

## Abstract

**Purpose:**

Data-driven harmonization can mitigate systematic confounding signals across imaging cohorts caused by variance in scanners and acquisition protocols. As diffusion magnetic resonance imaging data are often acquired with different hardware and software, harmonization is essential for integrating these scattered datasets into a cohesive analysis for improved statistical power. Large-scale, multi-site studies for Alzheimer’s disease (AD), a neurodegenerative condition characterized by high data variability and complex pathology, pose the challenge of both site-based and biological variation.

**Approach:**

We learn lower-dimensional representations of structural connectivity invariant to imaging cohort, geographical location, scanner, and acquisition factors. We design a conditional variational autoencoder that creates latent representations with minimal information about imaging factors and maximal information related to patient cognitive status. With this model, we consolidate 9 cohorts and 35 unique imaging acquisitions (for a total of 38 imaging “sites”) into a cohesive dataset of 6956 persons (16.4% with mild cognitive impairment and 10.7% with AD) imaged for 1 to 16 sessions for a total of 11,927 diffusion-weighted imaging sessions.

**Results:**

These site-invariant representations successfully remove significant (p<0.05) site effects in 12 network connectivity measures of interest and enhance the prediction of cognitive diagnosis (from 68% accuracy to 73% accuracy).

**Conclusions:**

The proposed model yields reproducible precision across 15 data configurations. This approach demonstrates the effectiveness of representation learning in enhancing biological signals by mitigating acquisition-specific confounding factors in neuroimaging studies.

## 1 Introduction

Changes in both the structure and function of the brain accompany the aging process, leading to a significant decline in cognitive abilities.^1^^–^^3^ Structural alterations contribute to deficits in functions such as multitasking, attention, memory, processing speed, and executive function.^1^^–^^3^ This decline especially impacts the 10.8% of Americans aged 65 and older affected by Alzheimer’s disease (AD) who experience a more rapid deterioration.^4^ The cognitive impairments that characterize AD, such as memory loss and difficulty with language, have been predominately associated with grey matter atrophy,^4^ but white matter, the brain’s communication network, undergoes several transformations, such as axonal degeneration, ischemia, and inflammation.^5^ Structural and connectivity alterations linked to AD begin to emerge up to 20 years before clinical symptoms are noticeable.^6^^–^^8^ Understanding these subtle, pre-symptomatic changes remains one of the major challenges in neuroscience.

Magnetic resonance imaging (MRI) is a non-invasive technique that employs strong magnetic fields and radio waves to create detailed images of internal bodily structures and tissues.^9^ Diffusion-weighted imaging (DWI), a specialized MRI technique, measures the self-diffusion of water molecules within tissues.^10^ Quantitative measures derived from DWI can be used to assess microstructural restrictions to water movement and microarchitectural integrity,^10^ which aid in the study of the underlying pathological changes associated with aging and AD.^2^^,^^11^^,^^12^ The use of advanced techniques, such as tractography and connectomics, allows for more comprehensive understanding of white matter tracks and neurobiological changes associated with neurodegeneration.^12^^–^^15^ Tractography is the process of reconstructing neural pathways (“streamlines”) from diffusion restriction.^16^ We approximate the whole brain with 10 million streamlines.^17^ We use the whole-brain reconstruction to create a structural connectome of the brain, which is the central focus of the field of connectomics. Briefly, a structural connectome is an adjacency matrix such that each matrix node is a defined grey matter region of interest (ROI).^18^ Edges represent the strength of the connection, such as the number of streamlines connecting two ROIs. The structural connectome indicates the brain regions connected via streamlines and quantifies the strength of those connections.^19^ We can then further describe the connectome using measures such as network disruptions, efficiency, modularization, connection strength, and centrality (i.e., connectome network measures) to deepen our study of neurodegeneration^19^

We hypothesize that these connectome network measures signal pre-symptomatic changes and characterize stages of cognitive decline. However, research conducted on the structural and network connectivity alterations associated with abnormal aging in patients with AD tends to be both less extensive and conducted with smaller sample sizes compared with studies on normal aging.^20^^–^^24^ Heterogeneous studies hinder the generalizability of findings to the larger population and compromise the establishment of consistent benchmarks.^25^ Hence, we must amalgamate smaller and scattered diffusion datasets specific to Alzheimer’s disease.^26^^–^^28^

**Fig. 1:**
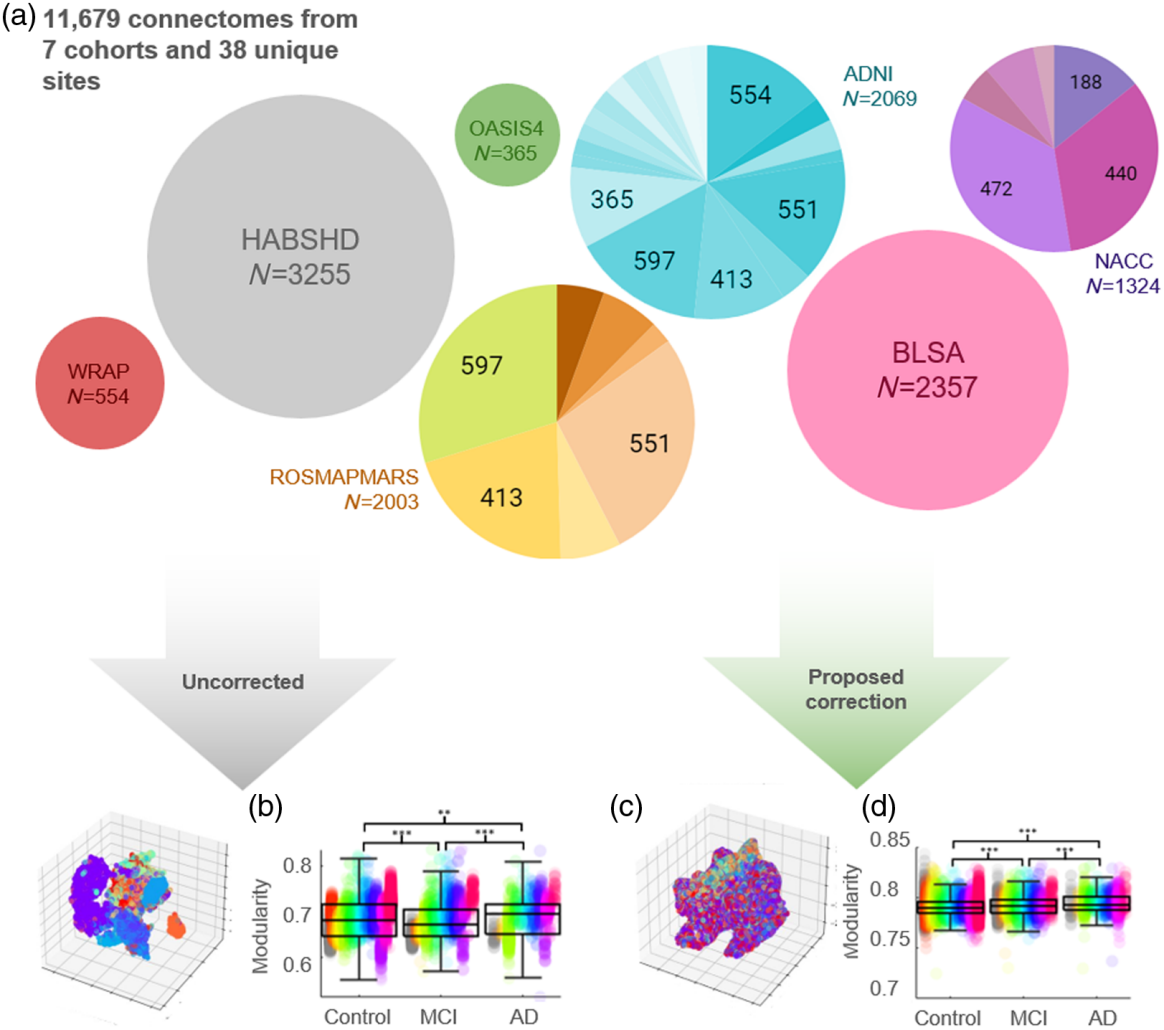
A total of 11,927 connectomes are pooled together across 9 cohorts and 35 unique imaging acquisitions (for a total of 38 “sites”). (a) If we naively combine all data and create *t*-SNE plots, there are obvious clusters driven by site. We compare cognitive diagnosis differences in one network connectivity measure, brain modularity, computed from these uncorrected connectomes. (b) Site-wise differences drive the detected differences and result in false findings. (c) In comparison, the *t*-SNE features of our learned representation have decreased site information, and the (d) resulting modularity values are harmonized and provide clear, significant (p<0.001) trends with cognitive diagnosis.

In this work, we combine connectomes from nine different cohorts: Baltimore Longitudinal Study of Aging (BLSA),^29^ Health and Aging Brain Study - Health Disparities (HABS-HD),^30^ Wisconsin Registry for Alzheimer’s Prevention (WRAP),^31^ Alzheimer’s Disease Neuroimaging Initiative (ADNI),^32^ Religious Orders Study (ROS),^33^ Rush Memory and Aging Project (MAP)33, Minority Aging Research Study (MARS),^34^ Open Access Series of Imaging Studies 4 (OASIS4), and National Alzheimer’s Coordinating Center (NACC).^35^ Within these cohorts, BLSA, ADNI, ROSMAPMARS, and NACC encompass images from multiple locations and scanner manufacturers (Fig. 1).

Multi-site diffusion imaging initiatives are the gateway to identifying meaningful, generalizable trends in normal and abnormal aging. However, these inter-cohort, inter-scanner, inter-location, and inter-acquisition factors introduce bias in DWI,^36^ subsequent models,^37^^,^^38^ and network properties.^39^^,^^40^ Naïve pooling of multi-site data leads to confounded effects and misleading results.^36^

Consequently, we must address site biases in network connectivity analysis through a process commonly known as “harmonization.”^36^ Harmonization is the application of statistical models, preprocessing, or machine learning to mitigate the effect of batches and sites.^36^ Effective harmonization reduces inter-site variation while preserving biological variation.^36^

Harmonization methods target different stages of diffusion-image processing and data types. In this work, we consider feature-based, image-based, and connectome-based. Feature harmonization methods correct site biases in scalars directly (i.e., fractional anisotropy and brain efficiency), typically as the final step before analysis. Image-based harmonization methods correct site biases in higher-order models of the diffusion-weighted signal (i.e., tensors and orientation distribution functions). Correction occurs during model fitting, prior to tractography or connectomics.^36^^,^^41^ Connectome-based harmonization involves synergizing connectome edge-weights for a pool of multi-site participants.^42^

ComBat, a feature-based harmonization method, is a widely used statistical tool initially designed for gene expression analysis and later extended to multi-scanner DWI harmonization.^36^^,^^43^ ComBat utilizes an empirical Bayes framework to estimate and correct multiplicative and additive site effects.^36^^,^^43^ Previous research indicates that ComBat effectively harmonizes differences between connectivity matrices and the graph measures themselves.^39^^,^^40^ Another common image harmonization technique, LinearRISH, estimates scanner effects in each DWI voxel.^44^ The target image’s scanner effects are projected to the reference image’s scanner effects. Previous study of these methods illustrates ComBat’s superior effectiveness and speed.^39^ Connectome harmonization has shown mixed results in previous works: Xu et al.^42^ concluded neuroComBat, mean shift, and CycleGAN style transfer methods did not yield consistent harmonization results. Onicas et al.^40^ reported similar findings in that parameter harmonization is superior to connectome-based harmonization.

However, recent assessments by Kim et al. highlight that ComBat’s accuracy relies on specific experimental design.^45^ Specifically, ComBat harmonization is well-behaved when analyzing associations with age, provided there is a cross-cohort matching (matching should be within 4 years and have at least 162 scans).^45^ ComBat shows significant limitations with minimal or no overlap among demographic covariates, such as age, sex, or disease status, whose effects we wish to maintain after site-specific harmonization.

We aim to harmonize learned connectome representations and use those synergized features to compute network properties of interest. We focus our efforts on harmonizing connectome features, a compromise between feature and connectome harmonization methods. The core technology is a site-conditional, variational autoencoder. By conditioning the latent space on unwanted imaging factors (i.e., “site”), the remaining features in the latent space learn to contain non-site information.^46^^,^^47^ In other words, we learn a set of site-invariant features of the connectome. We leverage these features to calibrate network connectivity measures for the purpose of studying AD progression. This conditional structure was first proposed for DWI by Moyer et al.^48^ and validated for use in connectomics in our previous work.^46^ We expand on the previously proposed model^46^ by generalizing the site-encoding mechanism, learning diagnosis-maximal features, and expanding analysis to 11,927 connectomes across 9 cohorts and 38 sites.

## 2 Methods

We learn representations of connectomes that are invariant to imaging site factors. Then, we show that these features produce network connectivity measures not biased by imaging site and are correlated with cognitive status. We compare our proposed method to ComBat harmonization for 15 data permutations.

### 2.1 Data

We source connectomes from nine major diffusion imaging cohorts: BLSA (955 persons, 2357 total sessions), HABS-HD (2610 persons, 3255 total sessions), WRAP (339 persons, 554 total sessions), ADNI (681 persons, 2069 sessions), OASIS4 (365 persons, 365 total sessions), NACC (1156 persons, 1324 total sessions), and ROS (77 persons, 123 total sessions), MAP (589 persons, 1570 total sessions), and MARS (184 persons, 310 total sessions) which make up “ROS/MAP/MARS.” Within these cohorts, BLSA, ADNI, ROS/MAP/MARS, and NACC encompass images from multiple locations and scanner manufacturers (Fig. 1). We consider ROS/MAP/MARS as a conglomerate because we consider the unique combination of imaging factors to be an individual site. Each site is encoded as a scalar 1 to *N*, where *N* is the maximum number of sites considered. Overall, the data consists of 61% females, 5069 (8857 sessions) persons with no cognitive impairment (NCI), 1142 persons (2053 sessions) with mild cognitive impairment (MCI), and 745 persons (1017 sessions) with AD. NCI, MCI, and AD designations are based on clinical diagnoses provided by each study. Full details on scanner, acquisition parameters, and locations for each site can be found in Supplementary Table 1. See Supplementary Tables 2 and 3 for the demographic details and diagnosis proportions for each site.

We define a site as a distinct combination of imaging location, scanner manufacturer, scanner model, and diffusion acquisition (TE/TR, number of shells, and number of directions collected). Further, we separate data by cohort. If a cohort provided site labels (i.e., ADNI, NACC, and ROS/MAP/MARS), we follow those designations.

### 2.2 Diffusion Processing

We correct all DWI for noise, motion, and eddy currents using the PreQual preprocessing tool.^49^ To model connectomes from DWI, we fit fiber orientation distribution functions to the signal in each voxel.^50^ Then, with probabilistic tractography (number of streamlines = 10,000,000,^51^ backtracking = true, seeding = grey matter white matter boundary region^17^^,^^52^), we create a whole brain tractogram for each scan.^53^ Combining the tractogram with 121 SLANT^54^ grey matter ROIs, we construct the connectome with 121 nodes and edges weighted by the number of streamlines connecting ROIs. In summary, the 11,927 connectomes are generated by assigning streamlines to grey matter ROIs based on the streamline endpoints. The connectome edge is defined by the total count of streamlines connecting two nodes. We normalize the connectome matrix to be between 0 and 1 before being passed through the harmonization model.

All data processing was done using the procedure outlined in Kim et al.^55^ A team of experts quality controlled each step of processing: DWI quality, PreQual correction, SLANT regions of interest, connectomes, and network properties with the ADSP_AutoQA tool.

### 2.3 Connectome Network Measures

We compute 12 network measures of interest using the Brain Connectivity Toolbox (version 0.6.1)^19^ corrected for streamline count.^45^ Complex network measures quantify functional segregation (clustering coefficient), modular structure (modularity), resiliency (assortativity), functional integration (global and local efficiency, path length, and edge count), and centrality (participation coefficient and betweenness centrality).^19^ In addition, we report basic network measures of node strength, density, and rich club coefficient.

The clustering coefficient describes the degree to which a node’s neighbors are also connected to each other.^19^ It is computed as a geometric mean and reflects the local cohesiveness of the network. Modularity refers to how well a network can be divided into non-overlapping communities or subgroups.^19^ It is a summary measure of the network’s community structure. Assortativity measures the tendency of nodes to connect with other nodes that are similar in some way (e.g., degree).^19^ It is calculated as a correlation coefficient among connected nodes and provides insight into the network’s structural robustness and resilience. Path length represents the average of the shortest distances between all pairs of nodes in the network.^19^ In this context, path length is measured in millimeters, reflecting physical distances in brain networks. Edge count is the number of nodes in the path length. Global efficiency quantifies how efficiently information is exchanged across the entire network.^19^ It is defined as the average of the inverse shortest path lengths between all pairs of nodes. Local efficiency measures how efficiently information is exchanged within the immediate neighborhood of a node.^19^ It reflects the local interconnectedness of the network and how well information is preserved within small clusters. The participation coefficient quantifies how evenly a node’s connections are distributed across different modules, indicating the diversity of its intermodular links.^19^ Betweenness centrality measures how often a particular node lies on the shortest paths among other nodes. It reflects the node’s importance in facilitating communication within the network.^19^ Node strength is the sum of the weights of all edges connected to a particular node, reflecting the total level of connectivity or influence of that node.^19^ Density is defined as the ratio of actual connections in the network to the total number of possible connections, providing a measure of overall connectivity.^19^ A rich club network is characterized by a core group of highly connected, central nodes that preferentially connect with one another, forming an influential and densely interconnected subnetwork.^19^

### 2.4 Model Architecture

**Fig. 2:**
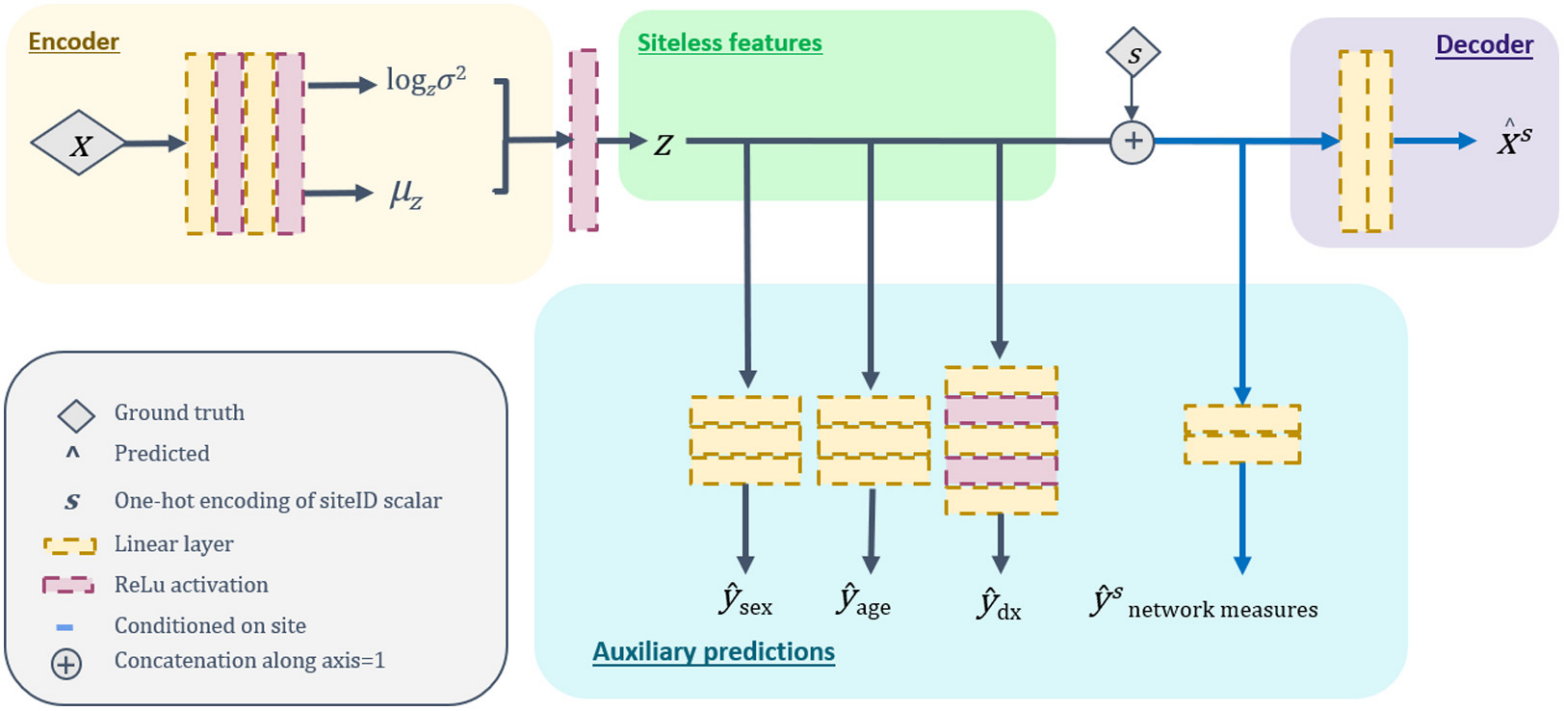
We implement a conditional variational autoencoder that is designed to learn a probabilistic mapping from an input connectome (x) to a latent vector z, conditioned on the *a priori* knowledge of which imaging site the connectome originated from (s). The model architecture is composed of an encoder (input dim = 121 × 121, output dim = 2 × 100), a latent space sampling mechanism (input dim = 200, output dim = 100), a decoder (input dim = 200), and several prediction layers for auxiliary tasks. The encoder consists of two fully connected layers followed by reparameterization (input dim = 121 × 121, output dim = 2 × 100), which samples from the variational Gaussian distribution defined by the learned mean ($\mu_{z}$) and variance ($\log\;\sigma_{z}^{2}$). The latent vector (dim = 100) is concatenated with a one-hot encoding (dim = 100) of the associated imaging site ID. Each connectome is reconstructed with a two-layer decoder (input dim = 200, output dim=121 × 121) (resulting in xs^). The model also produces auxiliary outputs: scalar patient age (y^age∈R), sex (y^sex∈{0,1}), diagnosis (y^dx∈{0,0.5,1}), and a vector of connectome network measures (y^snetwork measures∈R). The prediction of connectome network measures is also conditioned on imaging site, as we compare the predicted network measures to the network measures computed from the uncorrected connectome at site s. During the inference stage, we set s to be the target site for harmonization. As a result, the connectome network measures have a common site domain.

We developed a machine learning model that learns patterns in brain connectivity while accounting for differences among imaging sites (Fig. 2). The model takes a connectome as input and encodes it through several steps to create a compressed representation that also includes information about the imaging site where the data were collected. Using the learned latent vector, the model reconstructs the original connectome and predicts additional information, such as a person’s age, sex, diagnosis, and brain network measures.

#### 2.4.1 Theory

We designed the architecture such that the learned representation would have information relevant to the connectome, properties of network connectivity, participant age, participant sex, and participant diagnosis and limited information related to imaging factors (scanner hardware, scanner software, acquisition protocol, etc). These goals are translated to loss functions as follows.

Let x be the input connectome, x^ be the reconstructed connectome, and s be a one-hot site encoding. Each site (see Supplementary Table 1) is represented by a unique one-hot encoding. Under the conditional decoding structure, the variational posterior (probabilistic encoder) is the same as in the original variational autoencoder (VAE) model^56^

$$q(z|x)=N(z;\mu (x),\sigma (x{)}^{2}),$$ (1)

where μ(x) and $\sigma (x{)}^{2}$ are outputs of the encoder. We reparametrize z using the “reparameterization trick,”^56^ i.e.

$$z^{*}=\mu (z)+\varepsilon *\sigma (z),$$ (2)

where z differentiable with respect to μ and σ. The conditional decoder maps z to x conditional on s

(x|z,s)=x^. (3)

Overall, we optimize the autoencoder model by minimizing conditional reconstruction error, maximizing compression, minimizing conditional network measure prediction error ($L_{\mathrm{network}\_\mathrm{measures}}(s)$), and minimizing age ($L_{\mathrm{age}}$), sex ($L_{\mathrm{sex}}$), and diagnosis ($L_{\mathrm{dx}}$) prediction error. We weight each loss term to control the “importance” of the associated task with a coefficient a.

Together, the total loss is as follows:

$$L_{\mathrm{total}}=\alpha_{1}L_{\mathrm{recon}}(s)+\alpha_{2}L_{\mathrm{div}}+\alpha_{3}L_{\mathrm{age}}+\alpha_{4}L_{\mathrm{sex}}+\alpha_{5}L_{\mathrm{dx}}+\alpha_{6}L_{{\mathrm{network}}_{\mathrm{measures}}}(s).$$ (4)

Our reconstruction loss and *KL*-divergence equations follow those outlined in the VAE literature,^56^ with adjustments from Moyer et al.^47^^,^^48^ to optimize a site-conditional decoder. To derive the probabilistic encoder, we initially attempt to directly compute z given x. p(x|z) is the likelihood of the data (x) given latent variable z. p(z) is the prior distribution over *z*, which we assume to be a standard normal distribution. However, p(x) is an intractable distribution (i.e., there is an exponential lower bound on solving this equation). Therefore, instead of directly computing p(z|x), we approximate p(z|x) with q(z|x). We minimize *KL*-divergence between p(z|x) and q(z|x) (i.e., minimize differences in the two distributions). We simplify this minimization problem to become a maximization problem^47^

$$\max\;E_{q(z|x)}[\log p(x|z,s)]-KL[q(z|x)||p(z)],$$ (5)

such that the first term is the site-conditional reconstruction likelihood, $L_{\mathrm{recon}}(s)$ (computationally, reconstruction likelihood is mean squared error between x and $\hat{x}$). The second term is

$$L_{\mathrm{div}}=KL[q(z|x)||p(z)]=\frac{1}{2}\sum [\sigma^{2}(x)+\mu (x{)}^{2}-1-\log\;\sigma^{2}(x)].$$ (6)

We encourage the latent space to encode information related to participant age, diagnosis, sex and network connectivity properties by optimizing additional multi-layer perceptrons acting on the latent space (“auxiliary tasks” in Fig. 2). $L_{\mathrm{age}}$ [Eq. (7)] and $L_{\mathrm{dx}}$ [Eq. (8)] are mean squared error of predictions and demographic labels. $L_{\mathrm{sex}}$ is binary cross entropy [Eq. (9)]

Lage=1n∑(yage−y^age)2, (7)

Ldx=1n∑(ydx−y^dx)2, (8)

Lsex=1n∑ysexlog(y^sex)−(1−ysex)log(1−y^sex). (9)

$L_{\mathrm{network}\_\mathrm{measures}}(s)$ [Eq. (10)] is the mean squared error between the site-conditional network measures predicted from the latent space and the network measures computed from the original connectomes

Lnetwork_measure(s)=1n∑(ynetwork_measures(s)−y^network_measures(s))2. (10)

#### 2.4.2 Implementation details

For each data configuration defined in Supplementary Table 2, the proposed model is trained using the Adam optimizer for 2000 epochs with learning rate = $1\times 10^{-4}$ and mini-batches of 100 samples each. We train with data from sites with different sample sizes and demographics. To properly balance training, batches are pre-balanced with respect to site and cognitive diagnosis. We use a weighted random sampler to ensure each batch has the same proportions as the total dataset.

To determine the optimal set of hyperparameters for the proposed model, we conducted a systematic search over a predefined parameter space. The coefficient associated with the *KL*-divergence loss term, $\alpha_{2}$, is a key mechanism for controlling the regularization of the latent space. This hyperparameter was guided by the dual objective of maximizing site invariance while preserving predictive performance (network properties, age, sex, and diagnosis). To quantify site invariance, we measured the classification accuracy of site ID from the latent space.

### 2.5 Inter-Site Harmonization

First, we characterize the existing inter-site effects in the pooled uncorrected network measures. Then, we harmonize all 12 network measures with two different approaches: ComBat harmonization and our proposed method.

#### 2.5.1 Baseline 1: uncorrected

No correction is applied to connectomes or network measures.

#### 2.5.2 Baseline 2: ComBat harmonization

We compute the 12 network measures of interest from uncorrected connectomes. We correct these network measures using a MATLAB implementation of ComBat (v1.0.1, https://github.com/Jfortin1/ComBatHarmonization/tree/master/Matlab).^43^ ComBat is a feature-harmonization tool that corrects site-wise differences in mean and variance present in a set of scalars. We indicate that age, sex, and cognitive status should be preserved during harmonization.

#### 2.5.3 Proposed model harmonization

Using the trained model from Sec. 2.3, we replace the original site encodings with a common one. Therefore, we condition the reconstruction and network measures for all connectomes on the same site. The HABS-HD cohort is the largest dataset collected at a single-site, scanner manufacturer and acquisition protocols that we considered in this study. During inference, we condition each sample’s reconstruction and network measure prediction on the HABS-HD site encoding. The model’s auxiliary outputs are the 12 network properties of interest in the common site.

### 2.6 Performance Evaluation

The proposed machine learning model was evaluated to ensure it met two primary criteria: (1) reduction of site-specific systematic effects in the learned representations and derived network connectivity properties and (2) preservation of biologically meaningful information related to age, sex, and cognitive status. We conducted these evaluations at the level of the learned representation vectors and the computed network connectivity measures.

We evaluated if the representations contained site-specific information with a decision tree classifier to predict site labels from the learned representations and raw uncorrected connectomes. A lower classification accuracy indicated reduced site-dependence. In addition, we qualitatively assessed the representations of the three largest principal components with *t*-distributed Stochastic Neighbor Embedding (*t*-SNE) plots to examine whether clusters formed based on site.

To evaluate the biological relevance of the representations, we trained a decision tree classifier to predict cognitive status (NCI, MCI, or AD) with both the learned representations and the raw connectomes. We perform 10-fold cross-validation on this task and report the classification accuracies as mean and standard deviation across these folds. High classification accuracy indicated that the model effectively preserved cognitive status information in the representations.

We evaluated network connectivity measures for site invariance and biological consistency. We computed 12 network connectivity measures for three datasets: raw uncorrected connectomes, the site-invariant representations produced by the proposed model, and connectomes corrected using ComBat harmonization. To assess site effects, we applied a linear mixed-effects model to each network measure using the following equation:

$$y_{\mathrm{network}\_\mathrm{measure}}∼1+y_{\mathrm{age}}+y_{\mathrm{sex}}+y_{\mathrm{dx}}+s+s*y_{\mathrm{age}}+s*y_{\mathrm{sex}}+s*y_{\mathrm{dx}}+(1|\mathrm{Participant}),$$ (11)

where $y_{\mathrm{network}\_\mathrm{measure}}$ is one network measure: rich club coefficient, path length, average participation coefficient, average nodal strength, modularity, average local efficiency, global efficiency, edge count, density, average clustering coefficient, average betweenness centrality, or assortativity. Site ID, s, is encoded as a categorical variable. For persons with longitudinal data (e.g., data points that are not independent), we consider participant-specific random effects (“(1|Participant)”).

We compared the statistical significance and *F*-statistic of the coefficients related to site ID and its interactions across datasets. We consider a reduction in site-related effects in the network measures derived from the learned representations indicative of effective harmonization. The *F*-statistic is the ratio between the variation among sample means and the variation within the samples. We compute an *F*-statistic for each variable in Eq. (7). Inter-group differences due to site should not overshadow group differences due to diagnosis or sex. In other words, a reduction in site-related *F*-statistic and loss of statistical significance (p>0.05) are criteria for success.

Finally, we evaluated the robustness and generalizability of the proposed model by training it on 15 configurations of the data: one using all connectomes (38 sites) and 14 subsets in which 1 to 6 sites were systematically excluded. We examined the consistency of network connectivity measures across these training configurations to confirm the stability of the model’s performance under varying training data conditions. We evaluate precision with the coefficient of variation (CoV).^57^ Here, we compute CoV by dividing the standard deviation by the absolute value of the mean.^57^

## 3 Results

**Fig. 3:**
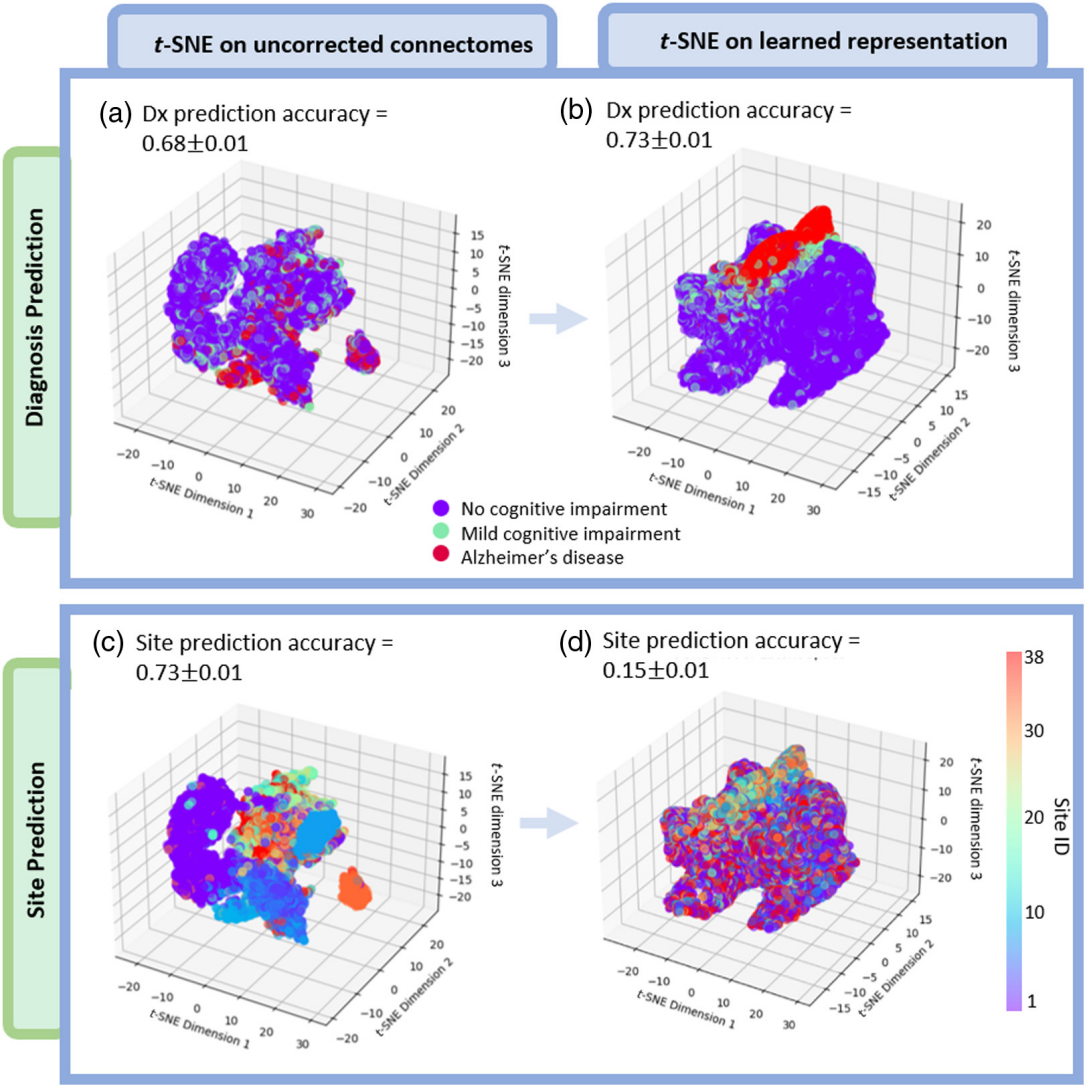
Our proposed model creates connectome representations that are invariant to imaging location, scanner, and acquisition, yet contains information related to cognitive diagnosis. We compare *t*-SNE representations for 11,927 uncorrected connectomes (a) and (c) and representations learned in our proposed model (b) and (d) for two tasks: encoding diagnosis (top) and site invariance (bottom). Separability driven by site decreases, and separability of diagnosis increases.

We compare representations curated by the proposed model to raw, uncorrected connectomes (Fig. 3). Cognitive status prediction accuracy improves from 0.68±0.01 to 0.73±0.01, and site prediction decreases from 0.73±0.01 to 0.15±0.01 accuracy. The progression from cognitively healthy, mild cognitive impairment, and, finally, AD is preserved and enhanced in the corrected feature space. Comparatively, site-wise clusters are not clear.

**Fig. 4:**
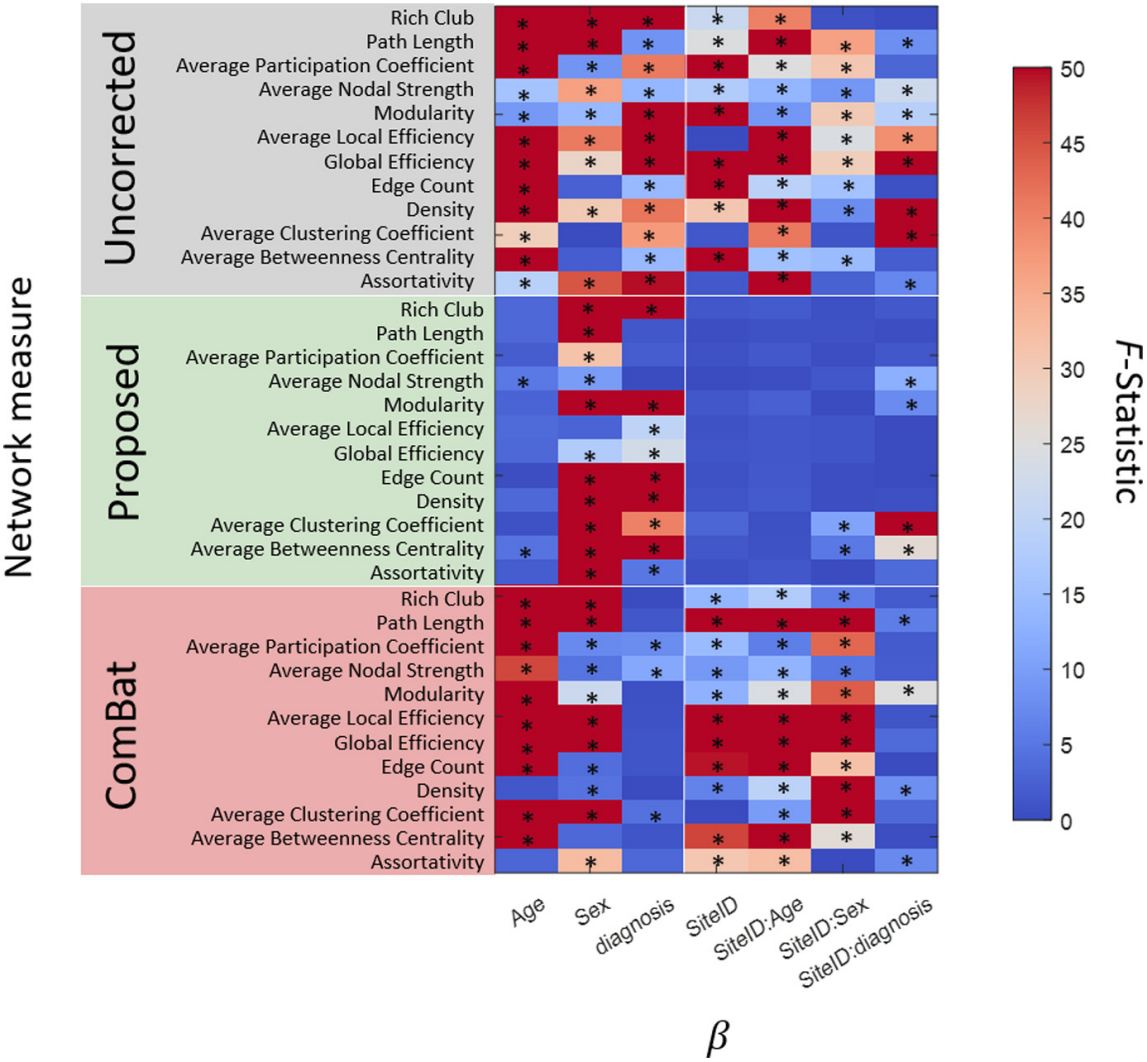
A total of 11,927 connectomes were pooled together. We compute network measures in the native imaging context (top), with our proposed model (middle), and ComBat (bottom). We fit a linear mixed effects model with coefficients of biological variables (age, sex, and diagnosis) and imaging site (site ID and site ID with each biological variable). *Significant coefficients (p<0.05). Then, we compare variance across these variables with analysis of variance (ANOVA) *F*-statistics and find that the proposed model enhances the detection of diagnosis and sex related differences and reduces imaging site discrepancies. ComBat does not reduce imaging site differences in all network measures. In particular, network measures related to traversing the network (path length, efficiency, and edge count) contain significant site effects after ComBat harmonization is applied.

Site-effects present in linear mixed effects model fits are removed in all 12 network measures corrected with the proposed model (Fig. 4). Similarly, the *F*-statistic of site with age, sex, and diagnosis decrease in all measures. The *F*-statistic is the ratio between the variation among sample means and the variation among all samples. For site-related variables, we expect less variation among site means. For diagnosis, we expect greater differences in NCI, MCI, and AD means. We therefore expect the *F*-statistic of site-related variables to be smaller than that of diagnosis variables. We observe decreases in site-related *F*-statistic and increases in diagnosis *F*-statistic in rich club coefficient, modularity, average local efficiency, global efficiency, edge count, density, average betweenness centrality, and assortativity. Significant site-related coefficients are removed in all except clustering coefficient, betweenness centrality, nodal strength, and modularity. *P*-values <0.05 associated with the categorical site variable are considered significant. After ComBat harmonization, the *F*-statistic of site-related effects decrease but remain significant in all except the clustering coefficient. In particular, network measures related to traversing the network (path length, efficiency, and edge count) contain significant site effects and large *F*-statistics after ComBat harmonization is applied.

**Fig. 5:**
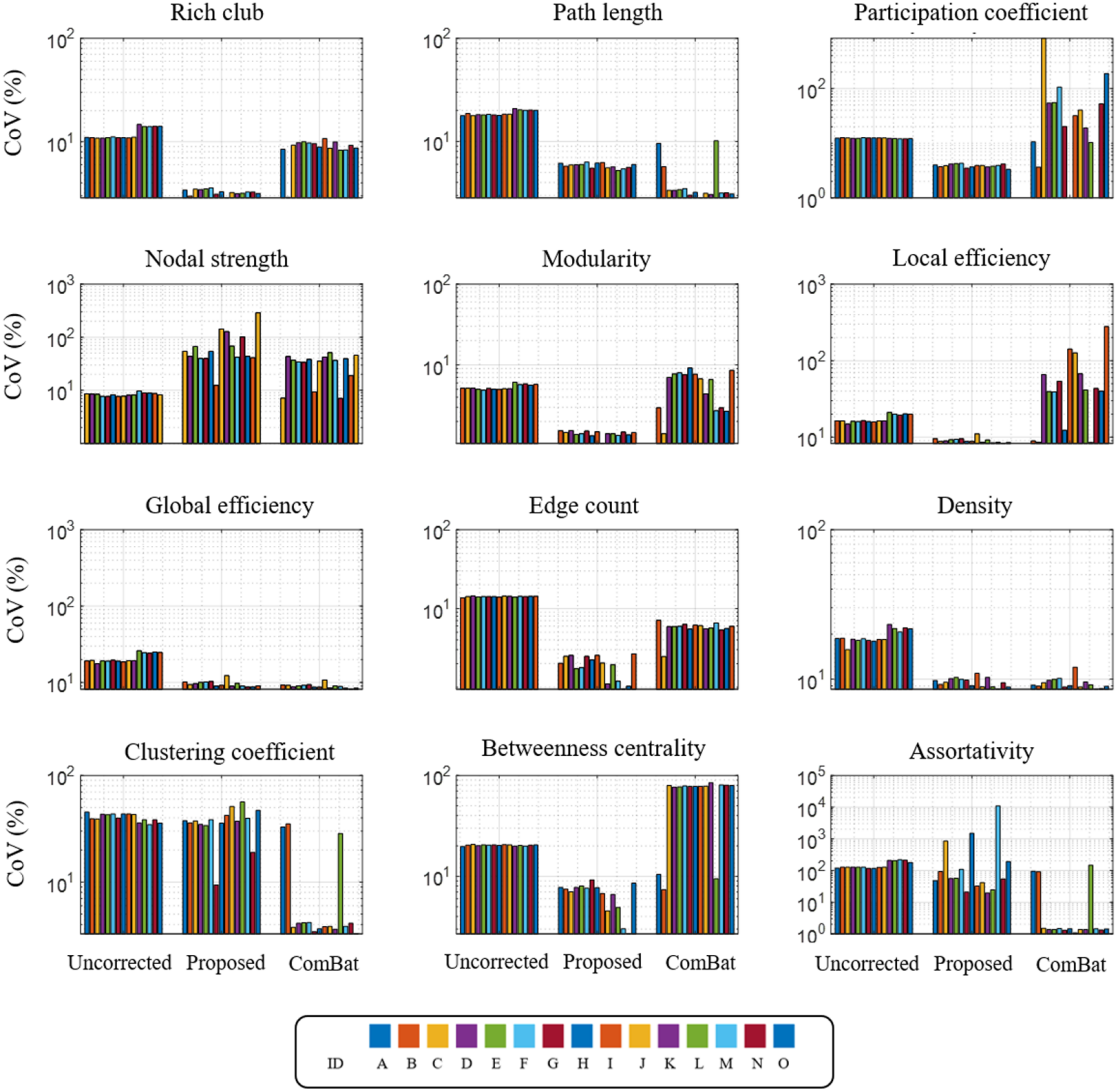
We examine the reproducibility across 15 data configurations (see Supplementary Table 2 for details on each configuration). We compute the CoV of network measures produced with the proposed model (“Proposed”), ComBat harmonization (“ComBat”), and the uncorrected baseline (“Uncorrected”).

We assessed the reproducibility of network measure values across 15 site-dropout scenarios (Fig. 5). CoV is a normalized estimate of precision.^57^ Ideally, harmonization increases precision in the pooled, multi-site data (i.e., comparable or lower CoV than the uncorrected network measures) and behaves consistently for different datasets (i.e., CoV is similar across datasets). The proposed correction consistently decreases CoV in all measures except for global efficiency, nodal strength, and assortativity. Notably, the proposed method decreases CoV more effectively than ComBat for the following network properties: rich club coefficient, modularity, edge count, local efficiency, participation coefficient, and betweenness centrality. Nodal strength and assortativity values corrected by the proposed method have mixed precision across datasets and are outperformed by ComBat.

Network measures computed for all 11,927 connectomes are shown in Supplementary Fig. 1. We group participants by cognitive status. Analyses of uncorrected network measures are confounded by inter-site differences. Although we observe significant differences in cognitive status, those findings are driven by site differences. Network measures corrected by ComBat have reduced site effects; however, we cannot detect significant differences associated with cognitive status in measures of functional integration (path length, global efficiency, and average local efficiency) (Supplementary Fig. 2). In network measures corrected by the proposed model, we find enhanced differences in each stage of cognitive decline (Supplementary Fig. 3) compared with baseline (Supplementary Fig. 1) and ComBat Harmonization (Supplementary Fig. 2).

Finally, we evaluated the reconstruction task by computing the difference between the connectomes constructed from site 1 DWI (native) and those reconstructed with the proposed method (reconstructed). We find that the reconstruction task achieved an error of 2212±61 streamlines on average across the connectome and 18±1 streamline error per node. Recall that each connectome was constructed with 10 million streamlines.

## 4 Discussion and Conclusion

The proposed infrastructure removed site-related effects in 11,927 connectomes originating from seven cohorts and 38 imaging parameters. We did not enforce age, sex, or diagnosis matching in the training data. In other words, we harmonize connectomes from heterogeneous sample sizes and demographics across an arbitrary number of sites (Supplementary Table 1). Our proposed model preserves network properties of functional segregation (clustering coefficient), modular structure (modularity), resiliency (assortativity), functional integration (global and local efficiency, path length, and edge count), centrality (participation coefficient and betweenness centrality),^19^ and basic measures of node strength, density, and rich club coefficient. We assert that these network measures characterize the stages of cognitive decline. We find significant group differences in cognitively unimpaired, mild impairment, and AD (Supplementary Fig. 2). The proposed model yields more robust biological findings than uncorrected or ComBat corrected measures.

For the measures of clustering coefficient and betweenness centrality, the model does not successfully remove site effects associated with diagnosis or sex. For the measures of nodal strength and modularity, the model does not successfully remove co-mingled effects of site and diagnosis. Although the additive effects due to site were ameliorated, the interaction effects of sex with site and diagnosis with site remain. We hypothesize that the proposed model may struggle to harmonize certain distributions. If the distribution of the network property differs greatly between cognitive status and the common site (site 1), we expect decreased performance.

Notably, there are few significant effects with age in corrected network measures (Fig. 4). Previous experiments by Wang et al.^58^ found minor changes in network measures with age. We reproduce the significant increase in nodal strength with age, and maintain that most network measures change very little with respect to age (less than 1% change per year).^58^ Clustering coefficient had the largest change per year with ∼4.2% increase per year, a finding we do not reproduce here. We observe that we do not completely ameliorate the interactive effects of site with sex and diagnosis from the clustering coefficient. In addition, we observe poor reproducibility for assortativity and nodal strength. We suggest using clustering coefficient, betweenness centrality, participation coefficient, assortativity, and nodal strength with caution.

In the case of LinearRISH^41^ or derived methods,^37^ there must be a subset of participants that have the same demographic profile. RISH methods are more appropriate in datasets with traveling heads, which may not be possible with a large number of diverse datasets. The proposed method does not require inter-site matched groups to calibrate harmonization. ComBat harmonization has a more relaxed inter-site matching criterion,^45^ however only operates on the final output scalars. We cannot tune harmonization to preserve or enhance relationships with covariates of interest. We find that with this limitation, ComBat is ineffective at capturing the relationships with cognitive status. Further, ComBat results in inconsistent precision across input data for studying modular structure (modularity), functional integration (edge count), centrality (betweenness centrality and participation coefficient), and rich club. In comparison, the learned feature vectors can be optimized for other covariates (i.e., handedness and race). The proposed model can be expanded to study more than the network connectivity properties studied in this work. In future work, we aim to harmonize and study local network properties for brain regions tied to memory and executive function.^1^^–^^3^

We acknowledge the following limitations of our study. We project all learned feature vectors to one common site, yet the choice of common site is non-trivial and potentially biases analysis.^37^ In future work, we propose projecting the learned feature vectors to a mid-space instead.^37^ Another key mechanic of our proposed method is to encode each site in a one-hot manner such that sites are treated as equally distinct. However, we note that in Supplementary Table 1, several sites share common scanner manufacturer, scanner model, field strength, and software. This more nuanced relationship cannot be captured in the one-hot encoding, which treats each site as equally distinct. In future work, we propose learning relative site embeddings to better capture these complex similarities and differences.

## Supplementary Material

10.1117/1.JMI.12.6.064001.s01

## Data Availability

Code is available at https://github.com/nancynewlin-masi/BrainNetworkHarmonization ADNI: Data used in the preparation of this article were obtained from the ADNI database (adni.loni.usc.edu). The ADNI was launched in 2003 as a public-private partnership, led by principal investigator Michael W. Weiner, MD. The primary goal of ADNI has been to test whether serial MRI, positron emission tomography, other biological markers, and clinical and neuropsychological assessment can be combined to measure the progression of MCI and early AD (https://adni.loni.usc.edu/). BLSA: https://blsa.nih.gov/ HABSHD: https://apps.unthsc.edu/itr/habs-hd NACC: https://www.naccdata.org/ OASIS4: https://sites.wustl.edu/oasisbrains/ ROS/MAP/MARS: https://www.rushu.rush.edu/research-rush-university/departmental-research/rush-alzheimers-disease-center/rush-alzheimers-disease-center-research/epidemiologic-research WRAP: https://wrap.wisc.edu/

## References

[1] Contemori G. Saccani M. S. Bonato M., “Multitasking effects on perception and memory in older adults,” Vision 6(3), 48 (2022). 10.3390/vision6030048 35997379 PMC9396999

[r2] Grieve S. M. et al., “Cognitive aging, executive function, and fractional anisotropy: a diffusion tensor MR imaging study,” Am. J. Neuroradiol. 28(2), 226–235 (2007). 17296985 PMC7977408

[3] Idowu M. I. Szameitat A. J., “Executive function abilities in cognitively healthy young and older adults—a cross-sectional study,” Front. Aging Neurosci. 15, 976915 (2023). 10.3389/fnagi.2023.976915 36845657 PMC9945216

[4] Rajan K. B. et al., “Population estimate of people with clinical Alzheimer’s disease and mild cognitive impairment in the United States (2020-2060),” Alzheimers Dement. 17(12), 1966–1975 (2021). 10.1002/alz.12362 34043283 PMC9013315

[5] Vernooij M. W. et al., “White matter microstructural integrity and cognitive function in a general elderly population,” Arch. Gen. Psychiatry 66(5), 545–553 (2009). ARGPAQ 0003-990X 10.1001/archgenpsychiatry.2009.5 19414714

[6] Jack C. R. et al., “Serial PIB and MRI in normal, mild cognitive impairment and Alzheimer’s disease: implications for sequence of pathological events in Alzheimer’s disease,” Brain 132(5), 1355–1365 (2009). BRAIAK 0006-8950 10.1093/brain/awp062 19339253 PMC2677798

[r7] Reiman E. M. et al., “Brain imaging and fluid biomarker analysis in young adults at genetic risk for autosomal dominant Alzheimer’s disease in the presenilin 1 E280A kindred: a case-control study,” Lancet Neurol. 11(12), 1048–1056 (2012). 10.1016/S1474-4422(12)70228-4 23137948 PMC4181671

[8] Villemagne V. L. et al., “Amyloid β deposition, neurodegeneration, and cognitive decline in sporadic Alzheimer’s disease: a prospective cohort study,” Lancet Neurol. 12(4), 357–367 (2013). 10.1016/S1474-4422(13)70044-9 23477989

[9] Westbrook C. and J. (Writer on magnetic resonance imaging) Talbot, “MRI in practice,” p. 395.

[10] Zhang J. et al., “Three-dimensional anatomical characterization of the developing mouse brain by diffusion tensor microimaging,” Neuroimage 20(3), 1639–1648 (2003). NEIMEF 1053-8119 10.1016/S1053-8119(03)00410-5 14642474

[11] Cohen A. A., “Complex systems dynamics in aging: new evidence, continuing questions,” Biogerontology 17(1), 205 (2016). 10.1007/s10522-015-9584-x 25991473 PMC4723638

[12] De Groot M. et al., “White matter degeneration with aging: longitudinal diffusion MR imaging analysis,” Radiology 279(2), 532–541 (2016). RADLAX 0033-8419 10.1148/radiol.2015150103 26536311

[r13] Hsu J.-L. et al., “Microstructural white matter changes in normal aging: a diffusion tensor imaging study with higher-order polynomial regression models,” NeuroImage 49(1), 32–43 (2010). 10.1016/j.neuroimage.2009.08.031 19699804

[r14] Lockhart S. N. DeCarli C., “Structural imaging measures of brain aging,” Neuropsychol. Rev. 24(3), 271–289 (2014). NERVEJ 10.1007/s11065-014-9268-3 25146995 PMC4163469

[15] Bookheimer S. Y. et al., “The lifespan human connectome project in aging: an overview,” Neuroimage 185, 335–348 (2019). NEIMEF 1053-8119 10.1016/j.neuroimage.2018.10.009 30332613 PMC6649668

[16] Conturo T. E. et al., “Tracking neuronal fiber pathways in the living human brain,” Appl. Phys. Sci. 96, 10422–10427 (1999). 10.1073/pnas.96.18.10422 PMC17904 10468624

[17] Smith R. E. et al., “Anatomically-constrained tractography: improved diffusion MRI streamlines tractography through effective use of anatomical information,” Neuroimage 62(3), 1924–1938 (2012). NEIMEF 1053-8119 10.1016/j.neuroimage.2012.06.005 22705374

[18] Shamir I. Assaf Y., “Tutorial: a guide to diffusion MRI and structural connectomics,” Nature Protocols 20(2), 317–335 (2024).1754-2189 10.1038/s41596-024-01052-5 39232202

[19] Rubinov M. Sporns O., “Complex network measures of brain connectivity: uses and interpretations,” Neuroimage 52(3), 1059–1069 (2010). NEIMEF 1053-8119 10.1016/j.neuroimage.2009.10.003 19819337

[20] Yin R. H. et al., “Multimodal voxel-based meta-analysis of white matter abnormalities in Alzheimer’s disease,” J. Alzheimer’s Dis. 47(2), 495–507 (2015). 10.3233/JAD-150139 26401571 PMC5757541

[r21] Xu S. et al., “The integrated understanding of structural and functional connectomes in depression: a multimodal meta-analysis of graph metrics,” J. Affect. Disord. 295, 759–770 (2021). JADID7 10.1016/j.jad.2021.08.120 34517250

[r22] Clerx L. et al., “New MRI markers for Alzheimer’s disease: a meta-analysis of diffusion tensor imaging and a comparison with medial temporal lobe measurements,” J. Alzheimers Dis. 29(2), 405–429 (2012). 10.3233/JAD-2011-110797 22330833

[r23] Yu J. Lam C. L. M. Lee T. M. C., “White matter microstructural abnormalities in amnestic mild cognitive impairment: a meta-analysis of whole-brain and ROI-based studies,” Neurosci. Biobehav. Rev. 83, 405–416 (2017). NBREDE 0149-7634 10.1016/j.neubiorev.2017.10.026 29092777

[24] Clerx L. et al., “P1-308: new MRI markers for Alzheimer’s disease: a meta-analysis of diffusion tensor imaging and a comparison with medial temporal lobe measurements,” Alzheimer’s Dement. 7(4S_Part_6), 405–429 (2011). 10.3233/JAD-2011-110797 22330833

[25] Smith S. M. Nichols T. E., “Statistical challenges in ‘big data’ human neuroimaging,” Neuron 97(2), 263–268 (2018). NERNET 0896-6273 10.1016/j.neuron.2017.12.018 29346749

[26] Wang T. et al., “Multilevel deficiency of white matter connectivity networks in Alzheimer’s disease: a diffusion MRI study with DTI and HARDI models,” Neural Plast. 2016(1), 2947136 (2016). 10.1155/2016/2947136 26881100 PMC4737469

[r27] Zhou Y. et al., “Hippocampus- and thalamus-related fiber-specific white matter reductions in mild cognitive impairment,” Cereb. Cortex 32(15), 3159–3174 (2022). 53OPAV 1047-3211 10.1093/cercor/bhab407 34891164

[28] Feng F. et al., “Altered volume and structural connectivity of the hippocampus in Alzheimer’s disease and amnestic mild cognitive impairment,” Front. Aging Neurosci. 13, 705030 (2021). 10.3389/fnagi.2021.705030 34675796 PMC8524052

[29] Ferrucci L., “The Baltimore Longitudinal Study of Aging (BLSA): a 50-year-long journey and plans for the future,” J. Gerontol.- Ser. A Biol. Sci. Med. Sci. 63(12), 1416–1419 (2008). 10.1093/gerona/63.12.1416 19126858 PMC5004590

[30] O’bryant S. E. et al., “The Health & Aging Brain among Latino Elders (HABLE) study methods and participant characteristics,” Alzheimer’s Dement.: Diagn. Assess. Dis. Monit. 13(1), e12202 (2021). 10.1002/dad2.12202 PMC8215806 34189247

[31] Johnson S. C. et al., “The Wisconsin Registry for Alzheimer’s Prevention: a review of findings and current directions,” Alzheimers Dement. 10, 130–142 (2017). 10.1016/j.dadm.2017.11.007 PMC5755749 29322089

[32] Jack C. R. et al., “The Alzheimer’s Disease Neuroimaging Initiative (ADNI): MRI methods,” J. Magn. Reson. Imaging 27(4), 685–691 (2008). 10.1002/jmri.21049 18302232 PMC2544629

[33] Bennett D. A. et al., “Overview and findings from the religious orders study,” Curr. Alzheimer Res. 9(6), 628–645 (2012). 10.2174/156720512801322573 22471860 PMC3409291

[34] Bennett D. A. et al., “The Rush Memory and Aging Project: study design and baseline characteristics of the study cohort,” Neuroepidemiology 25(4), 163–175 (2005). NEEPD3 0251-5350 10.1159/000087446 16103727

[35] National Alzheimer’s Coordinating Center, https://naccdata.org/ (accessed 29 Aug. 2023).

[36] Pinto M. S. et al., “Harmonization of brain diffusion MRI: concepts and methods,” Front. Neurosci. 14 (2020). 10.3389/fnins.2020.00396 PMC7218137 32435181

[37] Newlin N. R. et al., “MidRISH: unbiased harmonization of rotationally invariant harmonics of the diffusion signal,” bioRxiv, Cold Spring Harbor Laboratory Preprints (2023). 10.1016/j.mri.2024.03.033 PMC11283839 38537892

[38] Schilling K. G. et al., “Fiber tractography bundle segmentation depends on scanner effects, vendor effects, acquisition resolution, diffusion sampling scheme, diffusion sensitization, and bundle segmentation workflow,” Neuroimage 242, 118451 (2021). NEIMEF 1053-8119 10.1016/j.neuroimage.2021.118451 34358660 PMC9933001

[39] Newlin N. R. et al., “Comparing voxel- and feature-wise harmonization of complex graph measures from multiple sites for structural brain network investigation of aging,” Proc. SPIE 12464, 124642B (2023). PSISDG 0277-786X 10.1117/12.2653947 PMC10139749 37123017

[40] Onicas A. I. et al., “Multisite harmonization of structural DTI networks in children: an A-CAP study,” Front. Neurol. 13 (2022). 10.3389/fneur.2022.850642 PMC9247315 35785336

[41] Mirzaalian H. et al., “Harmonizing diffusion MRI data across multiple sites and scanners,” Med. Image Comput. Comput. Assist. Interv. 9349, 12–19 (2015). 10.1007/978-3-319-24553-9_2 27754499 PMC5045042

[42] Xu H. et al., “Evaluation of mean shift, ComBat, and CycleGAN for harmonizing brain connectivity matrices across sites,” Proc. SPIE 12926, 129261X (2024). 10.1117/12.3005563 PMC11415266 39310215

[43] Fortin J. P. et al., “Harmonization of multi-site diffusion tensor imaging data,” Neuroimage 161, 149–170 (2017). NEIMEF 1053-8119 10.1016/j.neuroimage.2017.08.047 28826946 PMC5736019

[44] Mirzaalian H. et al., “Multi-site harmonization of diffusion MRI data in a registration framework,” Brain Imaging Behav. 12(1), 284 (2018). 10.1007/s11682-016-9670-y 28176263 PMC7548102

[45] Kim M. E. et al., “Empirical assessment of the assumptions of ComBat with diffusion tensor imaging,” J. Magn. Reson. 11(2), 024011 (2024). 10.1117/1.JMI.11.2.024011 PMC11034156 38655188

[46] Newlin N. R. et al., “Learning site-invariant features of connectomes to harmonize complex network measures,” Proc. SPIE 12930, 129302E (2024). 10.1117/12.3009645 PMC11364372 39220624

[47] Moyer D. et al., “Invariant representations without adversarial training,” arXiv:1805.09458 (2018).

[48] Moyer D. et al., “Scanner invariant representations for diffusion MRI harmonization,” Magn. Reson. Med. 84(4), 2174–2189 (2020). MRMEEN 0740-3194 10.1002/mrm.28243 32250475 PMC7384065

[49] Cai L. Y. et al., “PreQual: an automated pipeline for integrated preprocessing and quality assurance of diffusion weighted MRI images,” Magn. Reson. Med. 86(1), 456–470 (2021). MRMEEN 0740-3194 10.1002/mrm.28678 33533094 PMC8387107

[50] Jeurissen B. et al., “Multi-tissue constrained spherical deconvolution for improved analysis of multi-shell diffusion MRI data,” Neuroimage 103, 411–426 (2014). NEIMEF 1053-8119 10.1016/j.neuroimage.2014.07.061 25109526

[51] Newlin N. R. et al., “Characterizing streamline count invariant graph measures of structural connectomes,” J. Magn. Reson. Imaging 58(4), 1211–1220 (2023). 10.1002/jmri.28631 36840398 PMC10447626

[52] “(ISMRM 2010) improved probabilistic streamlines tractography by 2nd order integration over fibre orientation distributions,” https://archive.ismrm.org/2010/1670.html (accessed 23 June 2022).

[53] Tournier J. D. et al., “MRtrix3: a fast, flexible and open software framework for medical image processing and visualisation,” Neuroimage 202, 116137 (2019). NEIMEF 1053-8119 10.1016/j.neuroimage.2019.116137 31473352

[54] Huo Y. et al., “3D whole brain segmentation using spatially localized atlas network tiles,” Neuroimage 194, 105–119 (2019). NEIMEF 1053-8119 10.1016/j.neuroimage.2019.03.041 30910724 PMC6536356

[55] Kim M. E. et al., “Scalable quality control on processing of large diffusion-weighted and structural magnetic resonance imaging datasets,” PLoS One 20(8), e0327388 (2025). 10.1371/journal.pone.0327388 40748971 PMC12316263

[56] Kingma D. P. Welling M., “Auto-encoding variational Bayes,” in 2nd Int. Conf. Learn. Represent., ICLR 2014 – Conf. Track Proc. (2013).

[57] Shukla-Dave A. et al., “Quantitative Imaging Biomarkers Alliance (QIBA) recommendations for improved precision of DWI and DCE-MRI derived biomarkers in multicenter oncology trials,” J. Magn. Reson. Imaging 49(7), e101 (2018). 10.1002/jmri.26518 30451345 PMC6526078

[58] Wang Y. et al., “Longitudinal changes of connectomes and graph theory measures in aging,” Proc SPIE 12032, 120321U (2022). 10.1117/12.2611845 PMC9060568 35506128

[59] Alzheimer’s Disease Neuroimaging Initiative, Sharing Alzheimer’s research data with the world, https://adni.loni.usc.edu (2025).

[60] Data and Publications Committee, ADNI Steering Committee, Acknowledgement List for ADNI Publications, http://adni.loni.usc.edu/wp-content/uploads/how_to_apply/ADNI_Acknowledgement_List.pdf (2013).

[61] Foundation for the National Institutes of Health (FNIH), Home | FNIH, http://www.fnih.org

[62] Rush Alzheimer’s Disease Center (RADC), RADC Research Resource Sharing Hub, http://www.radc.rush.edu.

