# Supplementary material for: Harmonizing 10,000 connectomes: site-invariant representation learning for multi-site analysis of network connectivity and cognitive impairment
Source: J Med Imaging (Bellingham). 2025 Nov 5;12(6):064001. doi: 10.1117/1.JMI.12.6.064001 (PMC12594104; doi:10.1117/1.JMI.12.6.064001)
Supplement: Supplementary file 1 [file JMI_012_064001_SD001.pdf]

Supplementary Material

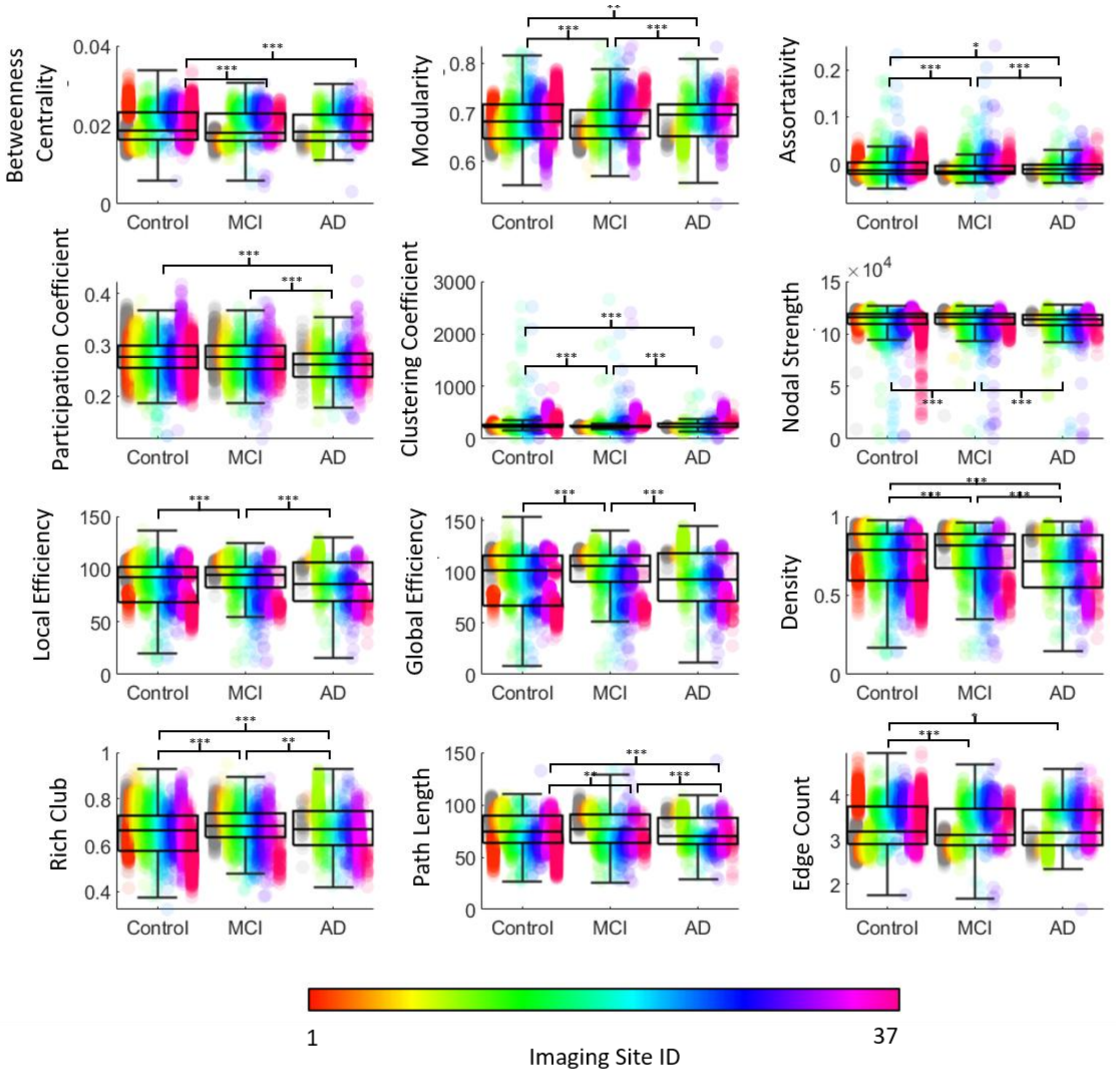

Supplementary Figure 1. We compute network measures for 11,927 connectomes in their native imaging site context and compare median values across diagnosis. We pool connectomes with common diagnosis and perform the Wilcoxon ranksum test of medians ("*" indicated $p < 0.05$, "**" indicates $p < 0.01$ and "***" indicates $p < 0.001$). Bias and inconsistent distributions between uncorrected imaging sites influence an analysis of diagnosis. See Supplementary Table 1 for Site ID descriptions.

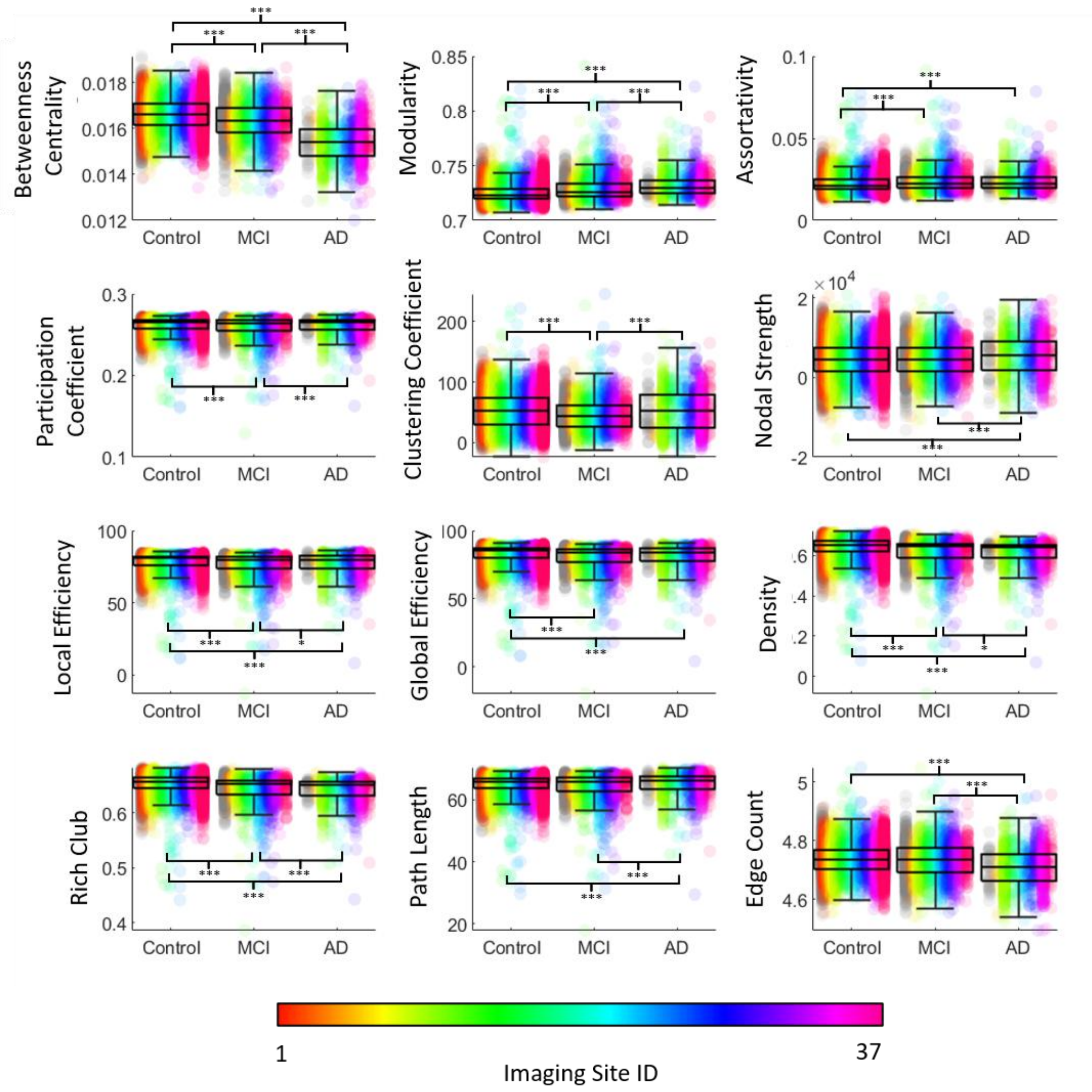

Supplementary Figure 2. We apply our proposed model to harmonize 11,927 connectomes and compare median values across diagnosis. We pool connectomes with common diagnosis and perform the Wilcoxon ranksum test of medians ("*" indicated p < 0.05, "**" indicates p <0.01 and "***" indicates p < 0.001). Additionally, we observe higher agreement in network measures across imaging site. See supplementary Table 1 for Site ID descriptions.

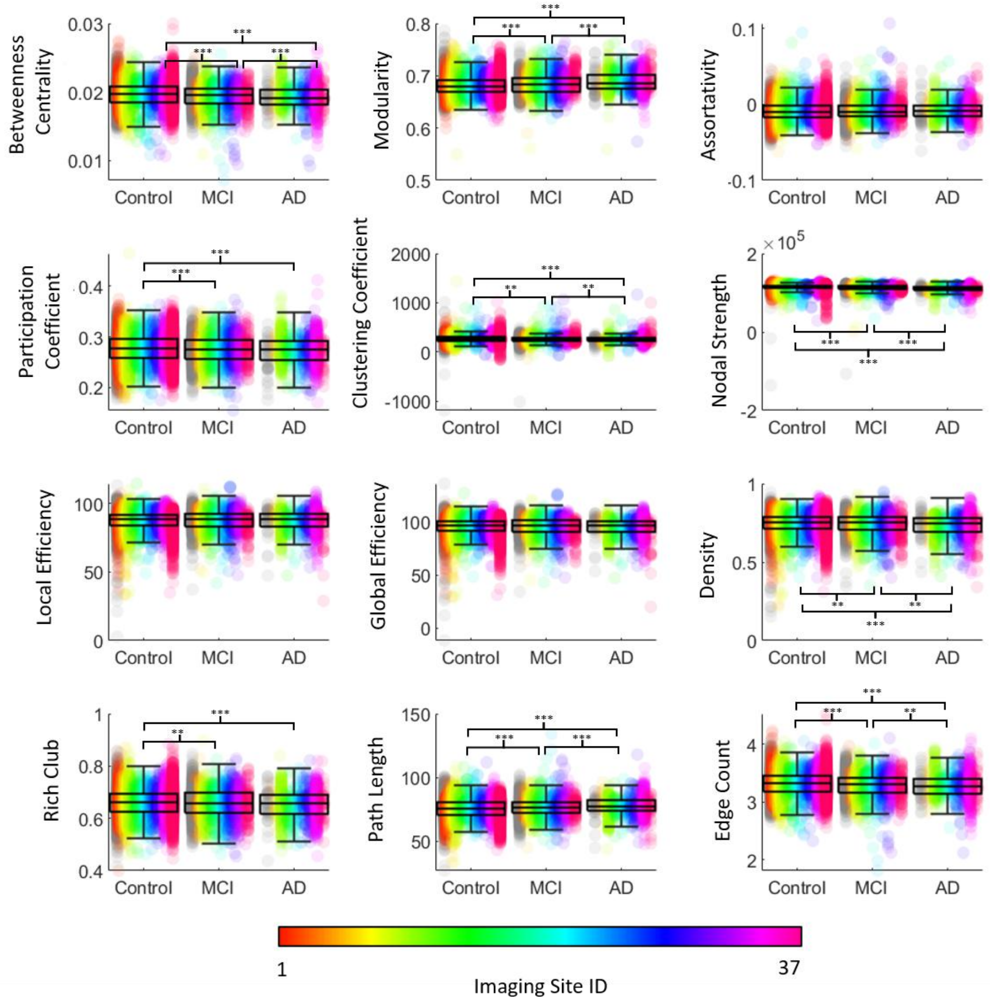

Supplementary Figure 3. We apply ComBat to harmonize 11,927 connectomes and compare median values across diagnosis. We pool connectomes with common diagnosis and perform the Wilcoxon ranksum test of medians ("*" indicated $p < 0.05$, "**" indicates $p < 0.01$ and "***" indicates $p < 0.001$. See supplementary Table 1 for Site ID descriptions.

Supplementary Table 1. Site lookup-table.

Site ID	Cohort Name	Location Code	# of Scans	TE (s)	TR (s)	# of directions (b=0)	Shells (s/mm^2)	Manufacturer	Model type	Field Strength	Site ID
**1**	HABSHD	UNT Healthcare	3255	0.09	4.0	204 (9)	0, 1000	Siemens	MAGNETOM Vida	3T	**14**
**2**	WRAP	Wisconsin Alzheimer's Institute	554	0.07	8.0	48 (8)	0, 1300	GE	DISCOVERY MR750	3T	**15**
**3**	ROS/MAP/MARS	University of Chicago BRIC_3T, uc_180604	111	0.05	11.5	41 (1)	0, 1000	PHILIPS-EKUR50U	Achieva_dStream	3T	**16**
**4**	ROS/MAP/MARS	University of Chicago BRIC_3T, uc_160125	138	0.05	11.0	41 (1)	0, 1000	PHILIPS-IJI1EMU	Achieva_dStream	3T	**17**
**5**	ROS/MAP/MARS	University_of_Chicago, uc_120221	51	0.05	12.0	41 (1)	0, 1000	PHILIPS-F398EB2	Achieva	3T	**18**
**6**	ROS/MAP/MARS	3T_Imaging_of_Morton_Grove, mg_160627	551	0.09	8.1	46 (6)	0, 1000	Siemens	TrioTim	3T	**19**
**7**	ROS/MAP/MARS	3T_Imaging_of_Morton_Grove, mg_160627	142	0.09	8.1	46 (6)	0, 1000	Siemens	TrioTim	3T	**20**
**8**	ROS/MAP/MARS	3T_Imaging_of_Morton_Grove, mg_120501	413	0.09	8.1	46 (6)	0, 1000	Siemens	TrioTim	3T	**21**
**9**	MAP	NLFH_Bannockburn, bnk_90211	597	0.09	5.4	84 (12)	0, 900	GE	SIGNA_EXCITE	1.5T	**22**
**10**	OASIS4	Washington University	365	0.10	3.8	21 (3)	0, 1000	Siemens	Skyra	3T	**23**
**11**	ADNI	99, Banner Alzheimers Institute	59	0.06	9.1	46 (5)	0, 1000	GE	DISCOVERY MR750	3T	**24**
**12**	ADNI	98, Lucas Center	73	0.07	9.1	46 (5)	0, 1000	GE	DISCOVERY MR750	3T	**25**
**13**	ADNI	941, Brown University	72	0.06	7.2	55 (7)	0, 1000	Siemens	Prisma_fit	3T	**26**

Cohort Name	Location Code	# of Scans	TE (s)	TR (s)	# of directions (b=0)	Shells (s/mm^2)	Manufacturer	Model type	Field Strength
ADNI	94, Brigham and womens hosp	80	0.07	12.5	46 (5)	0, 1000	GE	Signa HDxt	3T
ADNI	7, Mayo Clinic MRI 58	95	0.07	3.4	127 (12)	0, 500, 1000, 2000	Siemens	Prisma	3T
ADNI	6, UNIVERSITY of MICHIGAN Main	94	0.10	10.9	36 (4)	0, 1000	Philips	Ingenia	3T
ADNI	52, Sunnybrook Research Institute	68	0.07	3.4	127 (13)	0, 500, 1000, 2000	Siemens	Prisma	3T
ADNI	41, Yale University School of Medicine	58	0.06	7.2	55 (6)	0, 1000	Siemens	Prisma_fit	3T
ADNI	37, Indiana University	62	0.07	3.4	127 (13)	0, 500, 1000, 2000	Siemens	Prisma	3T
ADNI	3, USC Department of Radiology	140	0.07	13.0	46 (5)	0, 1000	GE	Signa HDxt	3T
ADNI	29, Newport Diagnostic Center 3T	81	0.08	15.3	36 (4)	0, 1000	GE	DISCOVERY MR750w	3T
ADNI	27, UIRR	221	0.06	7.8	54 (6)	0, 1000	GE	DISCOVERY MR750	3T
ADNI	21, Duke Univ Hosp MR5	120	0.06	7.8	54 (6)	0, 1000	GE	DISCOVERY MR750	3T
ADNI	168, Medical University of South Carolina	81	0.06	7.2	55 (6)	0, 1000	Siemens	Prisma_fit	3T
ADNI	16, MOUNT SINAI MEDICAL CENTER SKYRA 3T	115	0.08	9.6	55 (7)	0, 1000	Siemens	Skyra	3T
ADNI	135, Iowa MRRF	89	0.06	9.0	36 (4)	0, 1000	GE	Signa Premier	3T

Site ID	Cohort Name	Location Code	# of Scans	TE (s)	TR (s)	# of directions (b=0)	Shells (s/mm^2)	Manufacturer	Model type	Field Strength
**27**	ADNI	130, OSUMC Wright Center 3T 3-2955	70	0.09	10.1	33 (1)	0, 1000	Philips	Achieva dstream	3T
**28**	ADNI	129, Banner Alzheimers Institute	92	0.06	7.8	54 (6)	0, 1000	GE	Discovery MR750	3T
**29**	ADNI	127, WIMR	236	0.06	7.8	54 (6)	0, 1000	GE	Discovery MR750	3T
**30**	ADNI	126, WIMR	121	0.06	9.1	46 (5)	0, 1000	GE	Discovery MR750	3T
**31**	ADNI	109, Howard University	42	0.08	14.2	46 (5)	0, 1000	GE	Signa hdxt	3T
**32**	NACC	9661	188	0.10	8.8	65 (1)	0, 3000	Phillips	Achieva	3T
**33**	NACC	6518	440	0.09	8.0	26 (2)	0, 1000	GE	Genesis_sign a	1.5T
**34**	NACC	6499	472	0.08	8.0	48 (8)	0, 1300	GE	Discovery_m r750	3T
**35**	NACC	5783	75	0.10	9.5	92 (80)	0, 1000	Siemens	Triotim	3T
**36**	NACC	4032	106	0.06	8.0	26 (1)	0, 1000	GE	Discovery_m r750	3T
**37**	NACC	2578	43	0.06	9.1	7 (1)	0, 1000	GE	Discovery_m r750	3T
**38**	BLSA	10	2401	0.08	7.5	33 (1)	0, 700	Phillips		3T

Supplementary Table 2. Sites included in 15 training configurations

			Site ID																																						
**ID**	**# of scans**	**# of sites**	**1**	**2**	**3**	**4**	**5**	**6**	**7**	**8**	**9**	**10**	**11**	**12**	**13**	**14**	**15**	**16**	**17**	**18**	**19**	**20**	**21**	**22**	**23**	**24**	**25**	**26**	**27**	**28**	**29**	**30**	**31**	**32**	**33**	**34**	**35**	**36**	**37**	**38**
**A**	8,334	33	X		X	X	X	X	X	X		X	X	X	X	X	X	X	X	X	X	X	X	X	X	X	X	X	X	X	X	X		X	X	X	X	X		
**B**	8,438	33	X	X	X	X			X	X	X	X	X	X	X	X	X	X	X		X	X	X	X	X	X	X	X	X	X	X	X	X	X	X		X	X	X	
**C**	8,596	33	X	X	X	X	X	X	X		X	X		X	X	X	X	X	X	X		X	X	X	X	X	X	X	X	X	X	X	X	X		X	X	X	X	
**D**	8,832	33	X	X	X	X	X	X	X	X	X		X	X	X	X	X	X		X	X	X	X	X	X	X	X	X		X		X	X	X	X	X	X	X	X	
**E**	9,016	33	X	X	X	X	X	X	X	X	X	X	X			X	X	X	X	X	X	X	X		X	X	X	X	X	X	X	X	X		X	X	X	X	X	
**F**	9,134	33	X	X	X	X	X	X		X	X	X	X	X	X		X	X	X	X	X		X	X	X	X	X	X	X	X	X	X	X	X	X	X		X	X	
**G**	9,149	33	X	X	X		X	X	X	X	X	X	X	X	X	X	X	X	X	X	X	X		X	X		X	X	X	X	X		X	X	X	X	X	X	X	
**H**	9,154	33	X	X	X	X	X	X	X	X	X	X	X	X	X	X	X	X	X	X	X	X	X	X		X			X		X	X	X	X	X	X	X	X	X	
**I**	9,164	33	X	X		X	X	X	X	X	X	X	X	X	X	X			X	X	X	X	X	X	X	X	X	X	X	X	X	X	X	X	X	X	X		X	
**J**	9,571	37	X	X	X	X	X	X	X	X	X	X	X	X	X	X	X	X	X	X	X	X	X	X	X	X	X	X	X	X	X	X	X	X	X					
**J**	9,559	30	X		X	X			X	X		X	X	X	X	X	X	X	X		X	X	X	X	X	X	X	X	X	X	X	X		X	X		X	X		X
**L**	10,215	30	X		X	X			X	X		X	X	X	X	X	X	X	X		X	X	X	X	X	X	X	X	X	X	X	X		X	X		X	X		X
**M**	10,936	30	X	X	X	X	X	X		X	X	X	X				X	X	X	X	X		X		X	X	X	X	X	X	X	X	X		X	X		X	X	X
**N**	10,684	30	X	X	X	X	X	X		X	X	X	X				X	X	X	X	X		X		X	X	X	X	X	X	X	X	X		X	X		X	X	X
**O**	10,684	26	X	X			X	X	X	X	X	X	X	X	X	X			X	X	X	X		X					X		X		X	X	X	X	X		X	X

Supplementary Table 3. Demographic information for each imaging site

Site ID	Age (Mean ± Std)	# Scans	% Female	% MCI	% AD
1	65.62±8.59	3255	37.27%	20.25%	1.23%
2	62.55±6.75	554	35.20%	0.00%	0.00%
3	79.66±5.52	111	18.02%	9.91%	0.90%
4	78.36±6.40	138	15.22%	8.70%	0.72%
5	76.69±6.91	51	15.69%	9.80%	0.00%
6	82.55±6.92	551	20.15%	12.70%	2.36%
7	82.23±7.74	142	21.83%	11.97%	2.82%
8	81.76±7.74	413	22.28%	17.43%	0.48%
9	82.18±7.15	597	22.95%	18.59%	1.68%
10	74.92±8.87	365	47.67%	12.05%	75.62%
11	75.89±6.21	59	44.07%	32.20%	3.39%
12	75.70±6.09	73	72.60%	39.73%	20.55%
13	77.05±6.78	72	47.22%	19.44%	8.33%
14	70.30±6.22	80	57.50%	43.75%	17.50%
15	73.90±5.94	95	54.74%	40.00%	12.63%
16	74.88±7.25	94	72.34%	36.17%	8.51%
17	73.13±7.02	68	73.53%	66.18%	29.41%
18	74.00±7.45	58	58.62%	10.34%	5.17%
19	72.38±10.85	62	33.87%	27.42%	11.29%
20	73.08±7.72	140	44.29%	25.71%	14.29%
21	74.50±7.38	81	76.54%	40.74%	4.94%
22	74.76±7.20	221	54.75%	55.66%	11.76%
23	76.98±8.20	120	45.00%	41.67%	10.00%
24	75.15±7.44	81	35.80%	34.57%	19.75%
25	74.50±8.06	115	42.61%	41.74%	26.09%
26	73.12±5.82	89	55.06%	58.43%	11.24%
27	78.36±7.19	70	50.00%	42.86%	18.57%
28	73.78±6.95	92	42.39%	34.78%	10.87%
29	75.01±6.90	236	53.39%	27.12%	23.31%
30	73.07±6.32	121	67.77%	66.94%	24.79%
31	72.15±7.17	42	85.71%	88.10%	2.38%
32	73.80±7.76	188	40.43%	21.28%	17.02%
33	76.66±7.73	440	38.41%	13.18%	37.73%
34	64.07±10.14	472	41.10%	0.64%	23.73%
35	85.46±6.20	75	24.00%	5.33%	8.00%
36	73.40±5.79	106	37.74%	20.75%	11.32%
37	75.23±6.20	43	30.23%	9.30%	2.33%
